# Current Novel Caries Diagnostic Technologies: Restorative Dentists’ Attitude and Use Preferences

**DOI:** 10.3390/healthcare9101387

**Published:** 2021-10-17

**Authors:** Hani M. Nassar, Hanin E. Yeslam

**Affiliations:** Department of Restorative Dentistry, Faculty of Dentistry, King Abdulaziz University, Jeddah 21589, Saudi Arabia; hnassar@kau.edu.sa

**Keywords:** cariyes, enamel, early detection, restorative dentistry, preventive dentistry, fluorescence, transillumination

## Abstract

Early detection of caries lesions is key to a successful restorative dental treatment plan. The aim of this study was to investigate the preferences and attitude of graduate restorative dentistry residents (RDRs) regarding novel caries diagnostic technologies (NCDT) and to provide a brief overview of available technologies for both specialized and general dental practice. This cross-sectional study used an online questionnaire (17 questions) concerning RDRs’ attitude, preferences, and insights regarding five available NCDTs. It was distributed among twenty RDRs at a local government dental school following a review session about NCDTs. Collected responses were analyzed statistically using one-way analysis of variance (ANOVA), chi-squared with Bonferroni correction, and Kruskal-Wallis tests at a 0.05 significance level. Sixty-five percent of RDRs reported an interest in NCDTs as a discussion topic and almost half of them were positive towards their use, however, sixty percent of respondents were hesitant to diagnose caries solely using NCDTs. Fiber-optic-transillumination (FOTI) systems were ranked the best overall and with regard to all the investigated criteria (*p* < 0.05). Chosen reasons for FOTI included price followed by ease of use. In general, high price rated as the most perceived reason for not choosing a given NCDT followed by low practical applicability. Meanwhile, ease of use followed by relevant application ranked as the main reported reasons to choose an NCDTs.

## 1. Introduction

Dental caries is one of the most common wide-spread transmissible diseases affecting humanity [1,2,3]. It is a multifactorial disease characterized by subclinical dental tooth structure dissolution leading eventually to clinically detectable lesion formation. This is essentially the result of an imbalance in the oral cavity’s dynamic continuum of the demineralization and remineralization processes [4,5]. The earliest clinically visual manifestation of dental caries is known as white spot lesion (WSL) as it has a distinct chalky opaque appearance on enamel that is caused by a bacterial-acid-induced increase in the porosity of the superficial enamel layer [6,7]. At this stage and prior to frank cavitation of the tooth structure, several therapeutic measures can be applied to reverse or arrest the lesion [6,8,9].

Restorative treatment of the detrimental effects of dental caries in the oral cavity by merely replacing lost dental hard tissues takes a major toll on healthcare services worldwide [10,11,12]. Current dental practices aim at dealing with dental caries as a whole by focusing on prevention and possible reversal of the entire process, rather than merely managing the disease’s manifestations by artificially restoring resultant dental defects [8,13,14]. Even though the diagnosis of dental lesions is only one aspect of the disease’s management plan, rigorous procedures resulting in early detection and long-term monitoring of carious WSLs is paramount to its success [14,15,16,17]. Caries lesion detection, which basically refers to the identification of both early and established lesions by means of recognizing signs of altered physical and/or optical characteristics in the tooth structure [18,19] is traditionally accomplished by visual, tactile, radiographic examination, and/or a combination of all of them [14,20]. These examination techniques are also used for monitoring of the lesions. Caries monitoring usually refers to the evaluation of the signs of tooth structure alterations over time to depict whether the previously detected lesions were arrested or still progressing [19,21]. Monitoring can help with evaluating the success of caries preventive measures employed for the patient at hand [18]. Dentist’s skills and expertise along with the inherent challenging nature of early caries detection and diagnosis, proper risk assessment and effective monitoring of the lesions, all play a role in the eventual outcome of the restorative treatment plan [14,15,18]. Unfortunately, traditional tactile examination methods using a sharp instrument or the use of excessive pressure on the tooth structure may leads to cavitation of a non-cavitated early lesion [22,23]. Additionally, visual examination techniques can prove to be difficult to use when monitoring lesions over time, as it relies on the dentist’s abilities and subjectivity [24,25,26]. Moreover, radiographic examination for the detection and monitoring of the lesions would subject the patient to the harmful sequalae of radiation exposure [17]. Its diagnostic potential may also prove to be deficient as caries lesions might not be detectable prior to dentin involvement especially in the case of occlusal caries lesions (OCL), where most diagnostic techniques face challenges due to the anatomic and physical characteristics of the caries site under examination [27,28]. This is unfortunate given the reported difficulty of OCL prevention and management [29].

The dental practice is rapidly moving towards minimally invasive treatment modalities, which positively influence the development and introduction of several novel caries diagnostic technologies (NCDT) to facilitate early caries diagnosis leading to a possible end of the disease process to allow the utilization of effective remineralization preventive therapies [13,14,15,30,31]. Numerous studies investigated traditional diagnostic techniques (including visual and radiographic examination) and NCDTs with respect to their accuracy, sensitivity and specificity, and reliability in both early caries diagnosis and lesion monitoring. NCDTs’ effectiveness as an adjunct caries diagnosis method is well established in the literature [13,15,18,30,32]. Multiple NCDTs are currently available for dental practice and/or research, each with its own advantages, disadvantages, indications, and limitations. NCDTs rely on specific parameters to detect WSL and/or OCLs early and possibly monitor them reliably. Currently available CDTs include devices based on both heat and light [such as photothermal radiometry (PTR) and modulated luminescence (LUM)], transillumination [including both fiber-optic transillumination (FOTI) and digital optic transillumination (DIFOTI) using near infrared transillumination (NIT)], fluorescence [including quantitative light-induced fluorescence (QLF) and laser fluorescence (LF)] [14,15,26,33].

Despite the presence of numerous NCDTs on the market, their use amongst dentists is still limited [15]. This might be attributed to a deficiency, or lack thereof, in relative knowledge regarding NCDTs, their high price, dentists’ own skepticism, and/or fear of change [15,34]. Both traditional caries diagnostic methods and NCDTs are an integral part of graduate restorative dentistry training [35], thus one could argue that they are well established in a restorative dentist mindset. However, limited data is available regarding the actual use preference and attitude of restorative dentists towards NCDTs. The aim of the current study was to explore insights, device preferences, and attitudes of restorative dentistry residents towards five currently available NCDTs. Additionally, this paper aims at providing a summarized guide of novel technologies in caries diagnosis and monitoring to aid both general practitioners and restorative dentistry specialists. The null hypothesis of the current study was that there was no significant difference in RDR’s overall assessment and preference between the five presented NCDTs.

## 2. Materials and Methods

Ethical approval was obtained from the research ethics committee of the Faculty of Dentistry in King Abdulaziz University, IRB protocol 031-03-17.

**Figure 1:**
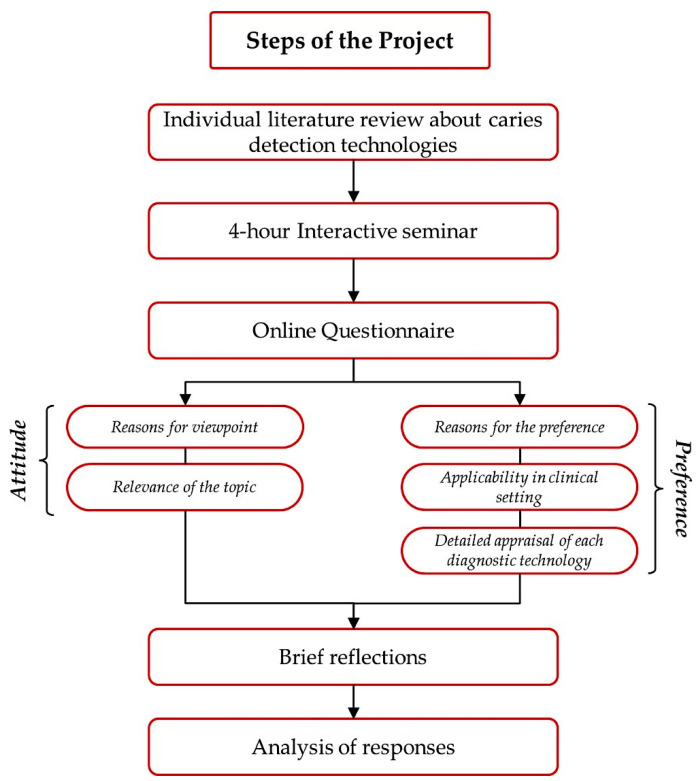
Flowchart showing the steps of the project.

This descriptive cross-sectional study was conducted using an online questionnaire that was formulated using Google Forms and was distributed amongst RDRs by sending them directly to their respective emails after an interactive theoretical session. The study’s objectives and goals were explained explicitly to all RDRs prior to their participation and consent was obtained thereafter. Participation and enrollment of RDRs was voluntary and all responses were anonymized to confirm confidentiality. Participating RDRs were exposed to traditional and novel CDTs as part of their graduate education. Additionally, they had to individually conducted a literature review of currently available novel CDTs and their related information and updates. Prior to participation in the survey, RDRs attended a 3 h-long interactive session that included a restorative-dentistry-consultant-lead objective recap of the various CDTs as well as a resident-lead dynamic discussion of their previously prepared literature reviews emphasizing their conclusions and insights, as well as the CDTs’ intricate details, including concepts, manipulation, indications, limitations, advantages, and disadvantages. Thereafter, a brief reflection regarding CDTs was prepared by each RDR. (Figure 1).

The questionnaire’s questions were formulated to assess attitude, opinion, and preference of restorative dentistry residents regarding NCDTs. They included questions concerning five commercially available NCDTs, namely: PTR and modulated LUM, FOTI/DIFOTI, QLF, LF, and NIT based devices. The questionnaire consisted of 17 questions in total; four multiple choice questions (MCQ), five Absolute Category Rating (ACR) scale questions that included five question items with the rating range from excellent to poor, seven questions using the 5-point Lickert scale and one free response format question. All questions were checked to establish both validity and reliability. The questionnaire was distributed amongst a group of restorative dentists with expertise in the field of caries diagnosis and the questions were adjusted accordingly. The 3 h interactive session prior to filling the questionnaire further ensured practicality and validity of the questionnaire. The average time needed to fill in the questionnaire was approximately 15 min.

The questionnaire began with an inquiry of the RDR’s demographic characteristics, then two Lickert scale questions addressed the participant’s opinion regarding the presented topic and their opinion regarding the potential benefit of NCDT’s use. Then the RDRs were asked to choose the preferred NCDT from the five previously presented categories. Afterwards, two MCQs assessed the respondents’ most likely perceived reasons for choosing an NCDT device and the main perceived reasons negatively affecting their NCDT choice. This was followed by five Lickert scale questions that aimed at assessing the participating RDRs’ attitude and interest in both NCDT’s use and further education. The remaining questions (ACR scale questions) aimed at rating the five previously discussed NCDTs in respect to five parameters, namely: ease of understanding the principle, practicality (ease of use, bulky device… etc.), rice, clinical application (caries detection, detecting cracks… etc.), and patient’s acceptance. Rating of each individual parameter for each NCDT was also included. The free response question including the RDR’s personal reflections qualitatively assessed the respondents’ opinions and insights regarding both presented NCDTs and other radiation-free caries diagnostic devices.

Statistical analysis of the results was conducted using SPSS computer software (Statistical Package for the Social Sciences, version 19.0, SPSS Inc., Chicago, IL, USA) at a significance level (*p* < 0.05). The received responses were tabulated and presented in the form of frequencies and percentages. Chi-squared statistical test was conducted to compare the five presented NCDTs, where each NCDT was rated according to five criteria; namely: ease of understanding, practicality (ease of use, bulky device… etc.), price, clinical application (caries detection, detecting cracks… etc.), and patient’s acceptance.

One-way analysis of variance (ANOVA) was conducted to detect significant differences in the overall assessment of the diagnostic aids between the groups. This was followed by Bonferroni test for multiple comparison between the responses rating the five presented NCDTs.

The Kruskal-Wallis inferential statistical test was conducted to rank the different NCDTs for each parameter with each parameter ranked individually to determine the preferred NCDT as reported by participating RDRs.

## 3. Results

All 20 RDRs that attended the revision session, agreed voluntarily to participate in the study and completed the questionnaire successfully resulting in a response rate of 100%. The participating RDRs population consisted of seven final year restorative dentistry residents, four residents in their third year of residency, six in their second year and only three first year residents.

**Figure 2:**
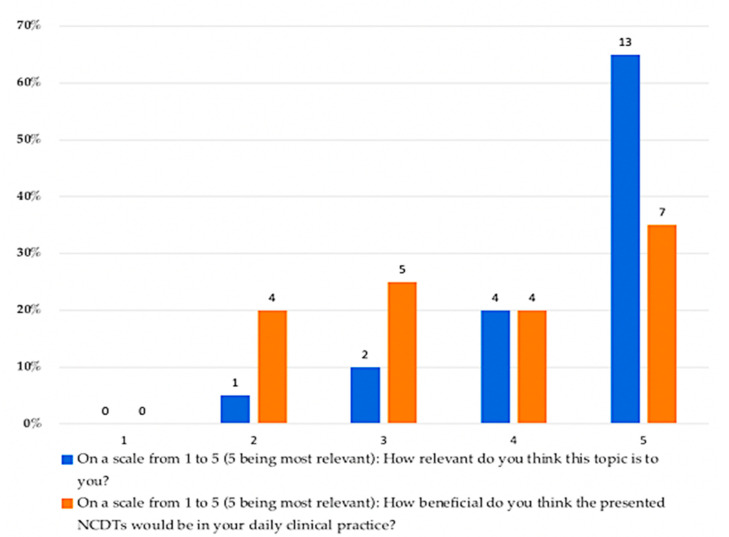
Percentages of the RDRs’ responses to the questions about their opinion regarding the relevance of NCDT as a discussion topic and how beneficial they think NCDTs would be in their clinical practice.

Regarding the RDRs opinion regarding NCDTs as a topic for further discussion, the majority of participating RDRs reported very high (13 (65%)) and high (4 (20%)) perceived relevance of NCDTs as a discussion topic. On the other hand, only two RDRs reported neutral responses, and one RDR reported a diminished perceived relevance of the topic. Regarding the RDRs opinion about the potential benefit of NCDTs in their own daily clinical practice, over half of them reported most beneficial (7 (35%)) and somewhat beneficial (4 (20%)), while a quarter of them were undecided on how beneficial the use of NCDT could be in their daily practice. Figure 2 shows the percentages of the responses to the questions regarding the RDRs opinion about the relevance of NCDT topic and the potential benefit of NCDTs in dental practice.

**Figure 3:**
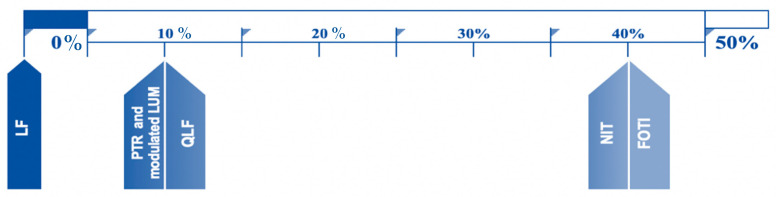
Percentage of RDRs responses when asked the following: Given all the factors discussed, including price, if you are given the choice for one diagnostic aid, which NCDT would you choose?

When RDRs were asked about their preferred NCDT choice when taking all previously reviewed and discussed factors into consideration, an equal number of RDRs chose FOTI and NIT devices (8 RDRs), followed by PTR with modulated LUM and QLF (2 RDRs). None of the responding RDRs LF as their preferred device. Figure 3 shows the percentages of RDRs responses to the question regarding the preferred choice of NCDT.

**Figure 4:**
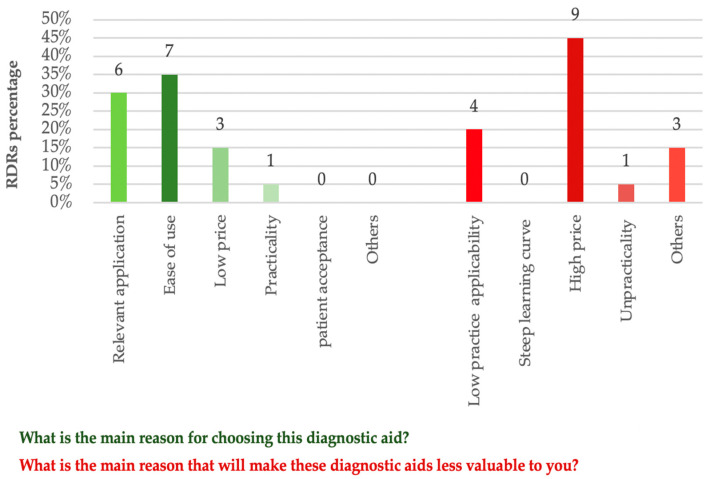
Percentages and frequencies of RDRs responses regarding the reason behind NCDT choice and the reasons making an NCDT less valuable to them as dental practitioners.

Regarding the reasons for choosing a specific NCDT, the vast majority of RDRs chose ease of use and relevant application as their main reasons (35% and 30%, respectively). On the other hand, when they were asked about their opinion regarding the main reason that would potentially cause an NCDT to be less valuable, almost half RDRs chose price as their main reason (45%). Three RDRs reported other reasons that were not included in the MCQ choices. They reported skepticism regarding the reproducibility, reliability, and specificity of an NCDT as other reasons to negatively influence their NCDT choice. Figure 4 shows the percentages and frequencies of RDRs responses to MCQs about the main reasons that would either positively or negatively affect their NCDT choice.

**Figure 5:**
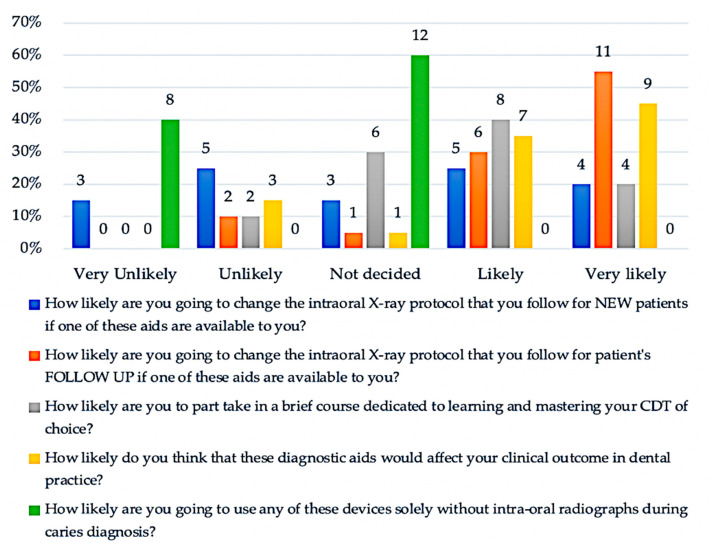
Frequencies and percentages of response to Likert-Scale questions regarding the likelihood of both the use of NCDTs in the RDRs’ restorative dental practice and their participation in NCDT-related brief educational activities.

Regarding the portion of the questionnaire aiming to assess the participating RDRs’ attitude and interest in both NCDT’s use and further education, the majority of RDRs reported a likely to very likely possibility (17 (85%)) of changing their current caries follow-up radiographic examination protocol if an NCDT was available in their clinic. However, less than half of them reported likely to very likely possibility of changing their current caries radiographic examination protocol when diagnosing new patients if an NCDT was available for use in their clinic. Furthermore, most RDRs were undecided when asked if they would completely rely on NCDTs to diagnose dental caries without intra-oral radiographs (12 (60%)). Despite this, almost half of the responding RDRs reported being completely against the exclusive use of NCDTs without radiographs during caries diagnostic procedures (8 (40%)), most of them reported a likely to very likely responses regarding the possible positive impact of NCDTs on the clinical outcome of their clinical practice (16 (80%)). Regarding the interest of the responding RDRs in NCDT-related further education, most of them reported likely very likely possibility of taking part in brief educational activities dedicated to improving their knowledge of their NCDT of choice (12 (60%)). Figure 5 shows the detailed frequencies and percentages of the RDRs responses to the Lickert-scale questions concerning their attitude towards- and preferences in- NCDTs use and their interest in NCDT further education.

**Figure 6:**
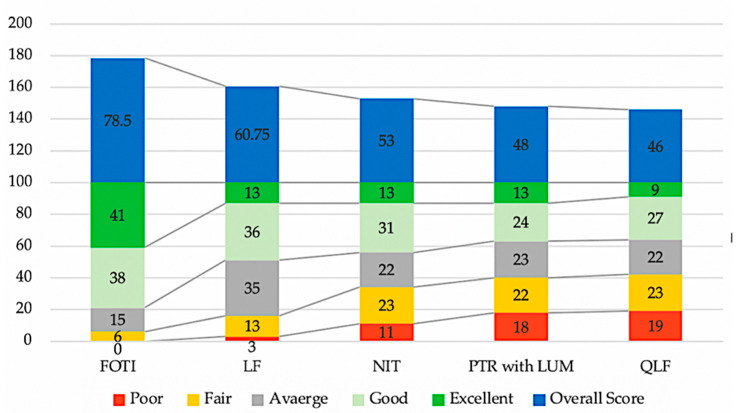
Ranking of NCDTs according to overall assessment (higher number indicates a better result).

Regarding the assessment of the five presented NCDTs, responses to the ACR questions portion of the questionnaire were analyzed using chi-squared test, where each NCDT was rated according to five criteria; namely: ease of understanding, practicality (ease of use, bulky device… etc.), price, clinical application (caries detection, detecting cracks… etc.), and patient’s acceptance. According to RDR’s responses in this section, FOTI ranked statistically significantly the best overall (chi squared *p*-value <0.001) as it received the highest number of excellent responses when counting all five criteria followed by LF (chi squared *p*-value <0.05). QLF had the lowest overall rank compared to the other NCDTs as it received more poor responses collectively, but the difference between QLF and PTR was statistically insignificant (chi squared *p*-value >0.05). Figure 6 summarizes the ranking of the five CDTs according to overall assessment of the RDRs responses to the ACR questions.

One-way comparison analysis of variance (ANOVA) showed that there is a statistically significant difference in the overall assessment regarding the afore mentioned criteria between the five NCDTs (*p* < 0.001). Table 1 shows the overall assessment of NCDTs using ANOVA.

Following the ANOVA results, the results of the multiple comparisons between the investigated NCDTs using Bonferroni correction method are detailed in Table 2. The results showed that FOTI is the best evaluated NCDT, and its score was statistically significantly higher than the other NCDTs (*p* < 0.05). Other NCDTs were not statistically significantly different when compared to each other (*p* > 0.05).

**Figure 7:**
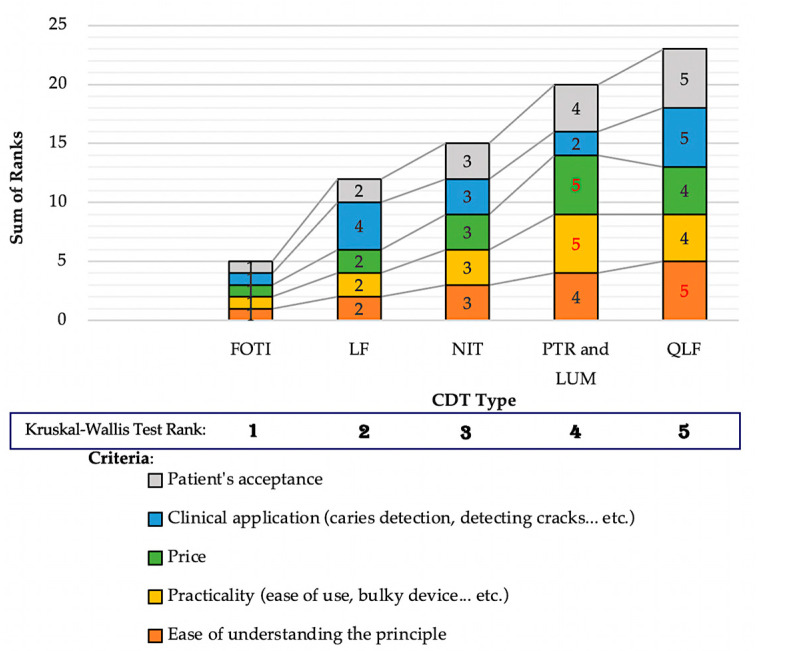
Sum of ranks of presented NCDTs for each of the five specified criteria arranged in order from best ranking (left) to worst ranking (right). The higher sum of ranks numbers indicates a lower ranked NCDT in respect to the investigated criterion.

Regarding the ranking of all NCDTs for each criterion, the sum of ranks for each NCDT according to each criterion was calculated, so that the NCDT with the lowest sum of ranks for each criterion would represent the best ranked NCDT. The null hypothesis of the Kruskal-Wallis statistical test comparing the mean ranks of the five NCDT groups was that the mean ranks of NCDTs were the same. The results showed that FOTI had the least sum of ranks across all investigated criteria, whereas QLF had the highest overall sum of ranks. Accordingly, FOTI was ranked the best reported NCDT and was statistically significantly different than the other NCDTs (*p* < 0.001). The results for the NCDT ranking for each criterion and respective Kruskal-Wallis rank are demonstrated in Figure 7.

The RDRs’ responses to the open-ended question disclosing their insights and preferences about NCDTs were mainly expressing hopes of wide-spread practical utilization of novel technologies in caries diagnosis and monitoring, especially the more objective NCDTs as an aid to traditional visual-tactile and radiographic examination procedures in daily dental practice. Half of RDRs expressed their belief in that successful application of such technologies still depends on the professional interpretation of the examination results and one RDR expressed skepticism regarding the reproducibility of the NCDT diagnostic results and their efficiency in monitoring carious lesions over time. A couple of responding RDRs also expressed their fear of over treatment, due to the higher sensitivity of NCDTs leading to the detection of more incipient lesions and some false positives along the way. Almost all RDRs reported the usefulness of NCDTs as an adjunct mean in caries diagnosis in their daily practice, especially in cases where access to the surface under investigation is difficult, and/or radiographic examination is not possible or not desirable. All RDRs reported the need for further clinical research facilitating an evidence-based applicable easy utilization of NCDTs in restorative dental practice as well in general dental practice. They additionally expressed their concern regarding the high price of some the NCDTs that could render them difficult to be used by dentists across the various parts of the healthcare system in the country.

## 4. Discussion

The primary objective of the current investigation was to investigate the preferences and attitude of graduate restorative dentistry residents with regards to novel caries diagnostic technologies. Based on the significant differences found, the null hypothesis was rejected.

Dental caries continues to be a prevalent disease affecting the global population [36]. Current dental practice is geared towards less invasive dental caries management by addressing the disease rather than just dealing with its detrimental effects using surgical/restorative approaches [13,14]. The widespread use of fluorides and early caries detection and remineralization therapies have reduced the negative impact of the disease on dental care services as many carious lesions remain as surface lesions rather than result in frank cavitation of the tooth structure [37,38].

Effective preventive dentistry requires accurate early detection and effective monitoring of surface lesions [14,17]. Assessing the detected lesion’s activity as well as its severity is both a difficult and extremely important task, as not all lesions would progress further [38]. Caries lesion activity assessments would lead to a more appropriate individualized preventive treatment modality [39]. One major problem facing practicing dental clinicians is the presence of hidden caries lesions that are difficult to detect and/or monitor with traditional clinical examination methods. Effective use digital technologies can help significantly in reducing the prevalence of hidden caries and thus more effective prevention of eventual cavitation [40]. This in addition to the need for less subjective methods of caries diagnosis has led to the development of enhanced caries diagnostic aids that include digital components and/or a sensor for better visualization and/or quantification of present lesions [24,33]. Despite the introduction of NCDTs and their successful use in research and literature supporting their beneficial clinical use, NCDTs are still seldomly used in daily practice or even covered in detail in dental schools [15,35]. Moreover, only limited information in the field of dental caries intricate diagnostic procedures is provided to dental students as part of their curriculum due to inter-faculty calibration challenges [41,42]. Despite the wide range of NCDTs types, current NCDT devices are based on one of five main categories. A summary of the five NCDT categories with their pertinent information is provided in Table 3.

Caries diagnostic devices and techniques have gone through numerous developments over the last decade [26]. This included efforts to increase their affordability, practicability, and availability to specialized and general dental practitioners. Unfortunately, clinicians still face a great difficulty in keeping up with continually developing methods and technologies available on the market worldwide [2,61]. Keeping track of each NCDT’s strengths and limitations, while assessing their possible usefulness in daily dental practice and trying to reach an evidence based informed decision is especially is very important [15] yet challenging with busy clinicians’ daily routines [32]. However, such difficulty does not seem to hinder the rising interest of dental practitioners in acquiring evidence-based intel regarding the newest caries detection and monitoring devices on the market [62,63]. This interest is noted in the current study, where most participating RDRs reporting their interest in NCDTs as a discussion topic. This can be attributed to the fact that such discussions can help clinicians in reaching well-informed decision when considering the appropriate updates to their usual caries diagnostic methods.

Detection of caries lesions as accurately and as early as possible is the foundation of current dental practice [14,15,26,32,53,64]. Unfortunately, this is a complex task that requires careful and methodical visual inspection of all tooth surfaces to detect subtle pathological changes in tooth tissues [18,32,33]. Visual caries diagnosis of the proximal surfaces is especially hard due to its obstructed view neighboring tooth and gingival tissues [30,31,53]. Regardless of the examination method chosen, whether traditional or novel, teeth must be free of plaque prior to examination [14]. Visual assessment with or without magnification aided by radiographic examination is considered the standard of caries diagnosis against which other methods are compared [15]. However, it is highly subjective and depends on the clinician’s visual acuity, clinical experience, and caries-related knowledge [18,26,34]. Several visual classification systems have been developed to classify dental caries lesions, including Nyvad, ICDAS, ADA CCS and several other systems. These mostly aimed at decreasing subjectivity and enabling incipient caries lesion monitoring [18,24,26,28,39,53,59,65]. NCDTs ease of use and practicality could enhance the clinician’s ability to make sense of the NCDT exam results as it compares to these visual classification systems allowing a more reliable communication regarding caries classification within the dental community [18,26]. Even though price was chosen as the main reason that would make an NCDT less valuable to and RDR Practicality and ease of use were chosen as the main reasons behind choosing a specific NCDT. Price was noted as a factor playing a role in the clinician’s choice of caries diagnostic device in a previous study [17] and could indeed be associated with dentists practicing in more affluent settings [34]. Unfortunately, there is not a single NCDT that fulfills all the desirable criteria for all clinicians to use them. However, the ease of use and short learning curve experienced by clinicians when using some of the new NCDTs, were reported in the literature as driving factors behind the continuous development of NCDTs [66,67,68].

Numerous reports in the literature supports the beneficial use NCDTs as adjuncts to visual examination aided by intra-oral radiographs, where indicated, and not to completely replace it [14,18,33,43,51,52,53,59,69,70]. This was also reflected in the current study, where more than half participating RDRs (55%) showed mostly positive responses when asked whether NCDTs are beneficial or not. The vast majority of RDRs in the current study accepted NCDTs as adjuncts to traditional caries diagnostic procedures and not as replacement to radiographs. This is consistent with the current conclusions and findings presented in literature. However, more recent studies have advocated the use of NIT devices and other NCDTs in daily caries diagnosis practice to avoid radiation exposure as much as possible [70,71]. Clinicians should be familiar with the limitations of each chosen method and use a comprehensive assessment, especially when formulating a treatment plan containing surgical restorative options [16,72,73]. The higher sensitivity of some NCDTs should not lead to an increase in the number of restorations and, rather, should be utilized in conjunction with visual examination to better monitor lesions’ behavior and confirm lesion presence [14,33]. This concern was also evident in the current study, where several RDRs expressed over treatment concerns when responding to the open-ended question regarding their NCDTs personal opinions. Since oral health improvement has been linked to a generalized improvement in body health [74], sensible monitoring of dental caries lesions aided with NCDTs is of prime importance in restorative dental clinics.

Most RDRs in the current study generally preferred FOTI as a caries diagnosis adjunct above other NCDTs. FOTI has been widely utilized, preferred [75], and investigated in the literature with numerous recommendations for its use as an adjunct to traditional diagnostic procedures [15,44,52,76]. The wider-spread use and use preference of FOTI compared to other devices might be attribute to the fact that it was one of the first technologies developed (in the late 1800s) [77] and therefore was more readily available and intensely investigated in the literature [51,52]. Numerous studies and literature reviews support the use of FOTI in daily clinical practice for both caries diagnosis and crack detection [52,78]. Current literature indicates the ease of use and diminished need for extensive experience or specialized training as advantages of FOTI as it optically increases the contrast between sound and carious tooth structure allowing visualization of the hidden lesion [14,44]. In the current study, RDRs ranked FOTI as the overall preferred NCDT having mostly excellent ratings. In a recent study, a qualitative assessment of FOTI indicated that it was welcomed by the participating clinicians who fancied its use as an additional tool for caries detection [78]. RDRs cited its relatively low price and ease of use as the main reasons for choosing it. However, the FOTI has some downfalls that are documented in previous publications but not mentioned by RDRs in the current study. These include the absence of an objective quantifiable means to measure the depth or assess the activity of the caries lesion, thus eliminating the possibility of longitudinal monitoring of the caries lesions [14,33,52,53]. Despite these disadvantages, studies have proven the effectiveness of FOTI in the detection of proximal caries lesions and is recommended in multiple studies for daily dental practice [26,76]. In the current study, FOTI was ranked statistically significantly better than all other NCDTs by participating RDRs. Laser fluorescence followed FOTI in rank with RDRs giving it a lower rating, especially regarding its clinical application. Studies support the use LF devices to confirm a questionable occlusal and proximal caries lesion, or when radiography is not applicable [43,54]. However, false positives are possible since the induced fluoresce is basically due to the bacterial porphyrins contaminating the dental tissue and the method was deemed not effective for secondary caries detection [79,80].

Even through NIT based NCDTs with longer wavelengths have improved lesion detection specificity along with imaging capabilities and is less affected by stains than other NCDTs, thus minimizing the chance of false positive results [48,60,69], its use is still limited comparably. This could be attributed to its very high price, which was chosen by most RDRs in the current study as the main reason to making a device less valuable in clinical practice. QLF in the current study mostly received poor rating; however, it was not statistically significantly different than LF, NIT, or PTR with LUM based NCDTs. One of the reported benefits of QLF in literature is its ability to measure both hard tissue changes as well as bacterial activity [14,45,46,81]. Newer QLF devices can analyze the entire oral cavity orally, however, it can be presumed that reported disadvantages such as high price, the need for training, and the steep learning curve are major hindering factors in daily dental practice [45,46]. This is also concurring with the current study’s responses. PTR with LUM based NCDTs are promising, with their ability to quantifying caries lesions using algorithm based numerical scale and its ability to detect lesions up to 5 mm below tooth surface in the presence of intact restorations [43,49,82]. However, its reported potential disconnect between the number generated by the device and significant clinical relevance might pose a major setbacks to its practicality and potential utilization by either general or restorative dentists [49], which is also reflected be the negative ratings given by RDRs to this NCDT as well.

## 5. Conclusions

Within the limitations of this study, it can be concluded that there is indeed an interest in the discussion, further education, and use of novel technologies by restorative dentists aiding in their caries diagnostic practices. When all factors are considered FOTI seems to be the preferred device amongst clinicians due to its affordability, ease of use, and practicality with possible additional uses in detection of other dental conditions such as cracks and fracture lines.

There is a noticeable trend in dentistry these days towards preventive dental approaches through early detection and effective monitoring of incipient caries lesions, especially amongst restorative dentists. However, the complexity and high price of some of these technologies are major factors working against their effective use in daily practice. Thus, providing further education to dentists harboring an interest in NCDTs can help better generalize their proper use. Further investigation into the NCDTs use preferences and insights of dental practitioners specializing in other fields of dentistry such as pediatric dentistry and advanced general dentistry might help shed more light on the subject and aid NCDT developers in improving the availability of NCDTs for general dental use, which eventually may lead to improved dental healthcare services across all fields of preventive dentistry.

## Figures and Tables

**Table 1:** Overall assessment of NCDTs using one-way analysis of variance ANOVA.

Overall Assessment of NCDTs Using ANOVA								
					95% Confidence Interval for Mean			
	N	Mean	SD *	SEM **	Lower Bound	Upper Bound	Minimum	Maximum
**PTR and modulated LUM**	20	48.00	16.65	3.72	40.21	55.79	20.00	80.00
**FOTI**	20	78.50	19.06	4.26	69.58	87.42	25.00	100.00
**QLF**	20	46.00	18.54	4.15	37.32	54.68	15.00	95.00
**LF**	20	60.75	15.92	3.56	53.30	68.20	25.00	85.00
**NIT**	20	53.00	21.11	4.72	43.12	62.88	20.00	85.00
One way Analysis of variance Table ANOVA								
		Sum of Squares		Df ***	Mean Square	F	P value	
**Between Groups**		13880.00		4	3470.00	10.31	0.000001	
**Within Groups**		31988.75		95	336.72			
**Total**		45868.75		99				

* Standard deviation (SD); ** Standard error of mean (SEM); *** Degree of freedom (Df).

**Table 2:** Shows the multiple comparison of NCDTs using Bonferroni method.

					95% Confidence Interval	
		Mean Difference	Std. Error	P Value	Lower Bound	Upper Bound
PTR and modulated LUM ^a^	FOTI	−30.5	5.8	0.000009 *	−47.18	−13.82
PTR and modulated LUM ^a^	QLF ^a^	2	5.8	1	−14.68	18.68
PTR and modulated LUM ^a^	LF ^a^	−12.75	5.8	0.304361	−29.43	3.93
PTR and modulated LUM ^a^	NIT ^a^	−5	5.8	1	−21.68	11.68
FOTI	QLF ^a^	32.5	5.8	0.000002 *	15.82	49.18
FOTI	LF ^a^	17.75	5.8	0.028869 *	1.07	34.43
FOTI	NIT ^a^	25.5	5.8	0.000289 *	8.82	42.18
QLF ^a^	LF ^a^	−14.75	5.8	0.126437	−31.43	1.93
QLF ^a^	NIT ^a^	−7	5.8	1	−23.68	9.68
LF ^a^	NIT ^a^	7.75	5.8	1	−8.93	24.43

Any two NCDTs having superscript ^a^ are statistically nonsignificant. *p*-values with * represents a statistically significant difference.

**Table 3:** Summary of the five currently available NCDTs on the market.

Parameter	Photothermal Radiometry and Modulated Luminescence (PTR/LUM)	Fiberoptic and Digital Fiberoptic Transillumination (FOTI and DIFOTI)	Quantitative Light-Induced Fluorescence (QLF)	Laser Fluorescence (LF)	Near infrared Light transillumination (NIT) and Near-Infrared Reflectance (NIR)
Commercial product examples	Canary System (Quantum Dental Technologies, Toronto, Canada)	Phatelus Optic Transillumination Light (NSK, Tochigi, Japan) Microlux (AdDent Inc., CT, USA) DiaLUX probe (KaVo Dental GmBH, Biberach, Germany)	* The Inspektor™ Pro QLF, * The Inspektor™ QLF-D Biluminator™ 2+ * Qscan™ (* Inspektor Research Systems BV, Bussum, The Netherlands)	DiagnoDent pen (KaVo Dental GmBH, Biberach, Germany)	DIAGNOcam (KaVo Dental GmBH, Biberach, Germany) CariVu (DEXIS, LLC, Hatfield, PA, USA)
Year of introduction *	2010	From 1990s	first generation Inspektor: 2004	Early 2000s	2012 (Europe) 2013 (USA)
Concept	Conversion of optical energy produced from a laser source into irradiation leading to temperature changes detected by an infrared detector [43]	White light scattering [14,34,44] (wavelength = 450–700 nm)	Green and red fluorescence of EDJ after exposure to visible blue light [45,46] (wavelength~400–488 nm)	Fluorescence due to bacterial protoporphyrin after the application of red light [14,26,43] (wavelength = 655 nm)	Transillumination of near- infrared light (wavelength ~780 nm) using two light emission windows [26,47] (NIR wavelength = 1310 nm) [48]
Main indication	Proximal caries lesions and cracks [49]	Detection of proximal caries lesions [14]	Smooth (facial) surface lesions [45]	Occlusal caries lesions [14]	Detection of proximal caries lesions in posterior teeth [50]
Caries lesion quantification **	Yes	No	Yes	Partial (0–99 scale depending on fluorescence of bacterial protoporphyrin) [51]	Partial (Grey scale)
Caries lesion activity determination [18,26]	No	No	Yes	No	No
Main shortcoming	It is often not possible to correlate the Canary Number to results of visual examination or bitewing (BW) radiographs [49]	No lesion quantification, Subjective [14,52], Potential higher false positive due to difficulty to differentiate between caries lesions and developmental defects and stains, False negative due to large restoration FOTI unsuitable for longitudinal lesion monitoring [53]	Not indicated for proximal lesions Sensitive to the ambient light, difficult accurate repositioning of intraoral camera type devices to take the next image [45]	High values for false positive, no detection of cavitation, no imaging [43], requires clean teeth and calibration on a sound surface before use [20,54]	Not indicated for smooth surface on the facial surfaces, No imaging of the caries extension relative to the pulp [50]
Main Advantage	Doesn’t require a dry field [51],detection of secondary caries around composite and below resin infiltrants, high repeatability. Canary Software connects with compatible practice management software, so that it can be easily moved around a dental office [49]	Affordability, short learning curve, Ease of use, applicability in other dental situations [52]	nondestructive quantification (numerical values) of the physical characteristics of caries lesions, QLF-D advanced device was able to analyze the entire oral cavity extraorally [26,45,46]	Caries monitoring possible [20,53]	NIR with longer wavelengths have no interference from occlusal surface stains [55], possible to detect caries lesions close to restoration margins, relative ease of use, crack detection, repeatability [50]
Set-up	Laser-emitting box with a handpiece containing an intraoral camera	Light-emitting electrode attached to a hand-held box (DiaLUXattached to the dental unit) (DIFOTI contains CCD sensor producing grey scale image)	Light box attached to a handpiece containing a light source and an intraoral camera	Red Laser producing box attached to a handpiece with different tips	Handpiece with near infrared light source and CCD sensor connected to a computer
Radiation/ Hazards *	Needs eye protection	None	Needs eye protection	Needs eye protection	None
Sensitivity (vs. X-ray ~0.41) [56]	range in 97% [49,57]	0.70 ± 0.01 to 0.50 ± 0.02 [58]	~0.69 [59]	0.76 to 1.00 [43]	99% for dentin caries detection on proximal surfaces under in vivo conditions [47] NIR:0.53 proximal and 0.49 occlusal [60]
Specificity (vs. X-ray ~0.78) [56]	range in 97% [49,57]	DIFOTI = 0.76	~0.83 [59]	0.47 to 0.94 [43]	NIR: 0.86 proximal and 0.70 occlusal [60]
Occlusal Caries **	Yes	No (error possibility)	Yes	Yes	Yes
Proximal caries **	Yes	Yes	No (detect bacterial porphyrins using 3rd generation QLF) [45]	Yes	Yes
Facial surface caries **	Yes	No	Yes	No	No
Price *	$$$$$	$	$$$$$	$$	$$$

* Estimate based on commercial products manufacturers-provided information, ** [14,33].

## Data Availability

The data presented in this study are available on request from the corresponding author.
